# Application of Object Detection Algorithms in Non-Destructive Testing of Pressure Equipment: A Review

**DOI:** 10.3390/s24185944

**Published:** 2024-09-13

**Authors:** Weihua Wang, Jiugong Chen, Gangsheng Han, Xiushan Shi, Gong Qian

**Affiliations:** 1 State Key Laboratory of Low-Carbon Thermal Power Generation Technology and Equipments, China Special Equipment Inspection and Research Institute, Beijing 100029, China; weihua0642@163.com (W.W.); 18262880256@163.com (J.C.); shixiushan@csei.org.cn (X.S.); qiangong@csei.org.cn (G.Q.); 2 China Special Equipment Inspection and Research Institute, Beijing 100029, China

**Keywords:** pressure equipment, non-destructive testing (NDT), object detection, deep learning

## Abstract

Non-destructive testing (NDT) techniques play a crucial role in industrial production, aerospace, healthcare, and the inspection of special equipment, serving as an indispensable part of assessing the safety condition of pressure equipment. Among these, the analysis of NDT data stands as a critical link in evaluating equipment safety. In recent years, object detection techniques have gradually been applied to the analysis of NDT data in pressure equipment inspection, yielding significant results. This paper comprehensively reviews the current applications and development trends of object detection algorithms in NDT technology for pressure-bearing equipment, focusing on algorithm selection, data augmentation, and intelligent defect recognition based on object detection algorithms. Additionally, it explores open research challenges of integrating GAN-based data augmentation and unsupervised learning to further enhance the intelligent application and performance of object detection technology in NDT for pressure-bearing equipment while discussing techniques and methods to improve the interpretability of deep learning models. Finally, by summarizing current research and offering insights for future directions, this paper aims to provide researchers and engineers with a comprehensive perspective to advance the application and development of object detection technology in NDT for pressure-bearing equipment.

## 1. Introduction

With continuous advancements in industrial technology, pressure equipment such as pressure vessels and piping plays a crucial role in sectors like chemical engineering, petroleum, and energy. The safety of these devices directly impacts the stability and sustainability of industrial production. However, due to prolonged usage and external environmental factors, these devices may develop various defects such as cracks, deformations, and corrosion, posing potential threats to their performance and safety. To promptly detect and address these issues, NDT technology has become one of the key methods to ensure the safe operation of pressure equipment [1,2,3,4,5,6].

Non-destructive testing (NDT) is a non-invasive method aimed at assessing and detecting defects within various materials and structures without compromising their integrity [4,6,7]. This technique plays a crucial role in inspecting defects in welded joints [8,9,10,11] and is pivotal across industries such as industrial materials [12,13,14], aerospace [15,16,17], healthcare [18], and pressure equipment testing [19,20,21], ensuring the safety and reliability of equipment and structures. However, employing NDT methods to detect defects often requires evaluating their location, shape, and size, making it a complex and labor-intensive task for inspectors. The ability of inspectors to detect defects is highly dependent on their accumulated experience with similar materials, necessitating professional expertise and proficiency. To enhance the speed, reliability, accuracy, and stability of inspections, there are increasing expectations within the industry for intelligent and user-friendly NDT equipment and instruments. With the advancement and dissemination of object detection technologies, the field of NDT is also witnessing new breakthroughs and opportunities. 

Traditional NDT methods, such as magnetic testing (MT), penetrant testing (PT), radiographic testing (RT), ultrasonic testing (UT), time of flight diffraction (TOFD), phased array ultrasonic testing (PAUT), magnetic flux leakage testing (MFL), eddy current testing (ECT), and acoustic emission testing (AE) have achieved certain accomplishments but still exhibit limitations such as complexity in operation, reliance on skilled professionals, and challenging working environments for inspectors. The rapid development of targeted detection technologies has brought new possibilities to NDT. Through techniques like image processing, pattern recognition, machine learning, and deep learning, automated analysis and identification of a large amount of data can be realized, thus improving the efficiency and accuracy of inspections [22,23]. 

By integrating NDT and object detection technologies, it becomes possible to achieve more accurate and faster detection and evaluation of defects within various materials and structures. This integration provides robust support for ensuring equipment safety and improving production efficiency, which aligns with the urgent industry needs for digitalization and intelligent development.

This paper will comprehensively review several classical algorithm models in the field of object detection technology and discuss the necessity of traditional and deep learning-based data augmentation methods in enhancing sample data for NDT technology. It will also analyze and organize research cases related to the joint use of NDT technology and object detection algorithms for identifying common defects in pressure equipment, such as pores, slag inclusions, cracks, corrosion, lack of fusion, and lack of penetration, aiming to improve the feasibility of intelligent and automated detection processes. Finally, the paper will provide an outlook on future research directions.

## 2. Defect Analysis of Pressure Equipment

Common defects in pressure equipment include surface defects, volumetric defects, and material structure defects. These defects may arise due to manufacturing processes, material quality, or improper operation, potentially affecting the equipment’s performance, safety, and durability. Therefore, using NDT methods to detect and assess these defects in a timely manner is crucial to ensure the proper operation and safety of pressure equipment. 

**Figure 1:**
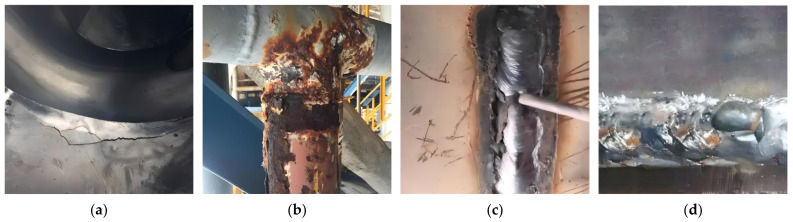
Several common types of surface defects: (**a**) surface cracks; (**b**) corrosion; (**c**) biting edges; (**d**) weld lumps.

Common surface defects in pressure equipment include cracks, dents, wear, corrosion, biting edges, and weld lumps. These defects can be caused by the prolonged use of the equipment, improper surface treatment, or uneven stresses during operation. Surface cracks may originate from thermal stresses during welding or fatigue due to long-term use in high-temperature and high-pressure environments. These cracks can not only reduce the mechanical strength of the equipment but also potentially propagate, leading to more severe damage in subsequent use. Dents and wear usually result from physical abrasion or improper operation. While these defects may seem minor on the surface, they can also become the starting point for corrosion. Corrosion, caused by chemical or electrochemical reactions, leads to gradual material loss from the equipment’s surface and poses a long-term threat to the structural integrity of pressure equipment. Figure 1 illustrates several common types of surface defects. These defects can affect the equipment’s sealing, mechanical strength, and durability, making timely detection and repair crucial. 

**Figure 2:**
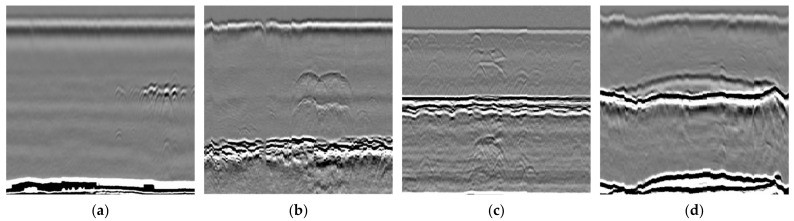
Several common types of volumetric defects: (**a**) inclusions; (**b**) crack; (**c**) lack of fusion; (**d**) lack of penetration.

Volumetric defects are primarily those internal to the material and often occur at the weld joints, potentially impacting the structural strength and stability of the equipment. These defects generally need to be detected using NDT methods such as UT, RT, or time of flight diffraction (TOFD). Common volumetric defects include porosity, inclusions, lack of fusion, and lack of penetration, which may be caused by uneven temperature distribution and inappropriate process parameters during casting or welding. Figure 2 illustrates the spectra of different defect types detected using TOFD. These defects can affect the material’s strength, toughness, and durability, making it essential to use appropriate NDT methods for timely detection and evaluation to ensure the safe operation of the equipment.

Material microstructural defects involve issues with the material’s microscopic structure, which can affect its performance and properties. Common types of microstructural defects include coarse grain size, uneven phase transformation, and incomplete annealing. These defects are typically caused by improper heat treatment or inadequate control during the manufacturing process. Coarse grain size can lead to decreased strength and toughness of the material, while uneven phase transformation can result in imbalanced physical properties, affecting the material’s pressure-bearing capacity. Microstructural defects are usually difficult to detect through conventional visual inspection, so detailed observation and analysis using methods such as metallographic microscopy or X-ray diffraction (XRD) are necessary to assess the material’s microstructure and implement necessary process improvements. 

By thoroughly understanding surface defects, volumetric defects, and microstructural defects in pressure-bearing equipment, one can more accurately assess the potential impact of these defects on the equipment’s performance and safety. Appropriate NDT strategies not only help in the early detection and evaluation of these defects but also effectively predict the equipment’s service life and implement preventive measures to ensure the reliability and stability of the equipment under high-pressure conditions.

## 3. Introduction to Object Detection Techniques

Object detection algorithms can be divided into traditional methods and deep learning-based methods. Traditional object detection algorithms are relatively simple, easy to understand, and implement. However, they have limited performance and generalization ability for complex scenes, diverse targets, and large-scale data. They struggle to adapt to different tasks and datasets, presenting several limitations. In contrast, deep learning-based object detection algorithms automatically learn features and patterns from images, exhibiting superior generalization ability and robustness. They perform well in large-scale data and complex scenarios, capable of handling targets with various scales, poses, and lighting conditions. With the rapid development and evident advantages of deep learning-based object detection algorithms, they have become a research hotspot among scholars and engineers in the field of object detection. Integrating with different task requirements, these algorithms have made significant advancements in intelligent surveillance and security, autonomous driving, medical image analysis, industrial quality inspection, and intelligent detection, fostering the emergence of new technologies and driving societal development [24,25,26,27,28,29,30,31,32,33].

In the field of object detection algorithms, convolutional neural networks (CNNs) [34] are widely used as the foundational network for feature extraction in various object detection algorithms. Classic object detection algorithms include Faster region-based convolutional neural network (Faster R-CNN) [35], You Only Look Once (YOLO) [36], and Single Shot MultiBox Detector (SSD) [37]. Although U-Net [38] is primarily used for medical image segmentation tasks, it is also employed in some NDT applications for image segmentation to detect material defects or anomalies [39,40,41,42,43].

### 3.1. CNNs

The development of CNNs began in the late 1980s and early 1990s, but it was not until 2012 that the success of the AlexNet model in the ImageNet image classification challenge brought significant attention and application to CNNs. CNNs are feedforward deep neural networks with convolutional structures. They can automatically learn signal features from images through forward propagation and adjust network parameters based on negative feedback. Training the network enables automatic optimization of the model. CNNs have found widespread applications in image recognition and classification [44,45], video recognition [46,47], natural language processing [48,49], visual tracking [50,51], and other fields. They are known for their excellent performance in processing image tasks [52]. 

**Figure 3:**
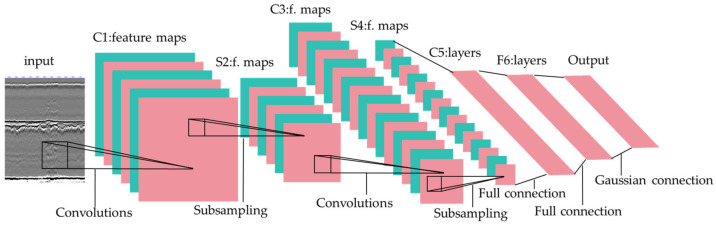
Architecture of CNNs. Adopted from [34].

The basic structure of a CNNs includes convolutional layers, pooling layers, activation functions, and fully connected layers. The core idea is to extract features from input data through convolution and pooling operations and then use these features for classification or regression tasks. The fundamental architecture is illustrated in Figure 3. 

The convolutional layer is used to extract features from input images. Convolutional operations involve sliding a convolutional kernel over input data, performing a weighted sum at each position to generate a feature map. Convolutional operations preserve spatial structure information, and through learned kernels, networks can automatically extract features from input data, such as edges and textures. The number of convolutional kernels in CNNs is positively correlated with the number of extracted image features. Convolutional operations enhance the characteristics of signals.

The pooling layer, also known as the subsampling layer, is used for dimensionality reduction and compression of image information. Pooling operations are typically employed to decrease the spatial dimensions of feature maps. Common pooling operations include max pooling and average pooling. Pooling layers help to extract spatial hierarchical features while reducing the model’s sensitivity to the exact spatial location of features, thereby enhancing computational efficiency. 

Activation functions play a crucial role in neural networks. By introducing non-linear characteristics, they enable neural networks to learn and represent complex non-linear relationships, thereby enhancing the model’s expressive power. Activation functions map input signals to outputs, helping the network effectively capture structures and patterns within data. Additionally, activation functions determine the activation state of neurons, controlling which neurons are active for specific inputs, thereby further influencing the network’s learning and representation capabilities. In terms of gradient propagation, appropriate activation functions can effectively mitigate issues such as gradient vanishing or exploding, thereby aiding in stabilizing and accelerating the training process of deep neural networks. Commonly used activation functions include ReLU (rectified linear unit), sigmoid function, and tanh function. 

The fully connected layer, also known as the dense layer, is typically used in the final layers of neural networks. It is responsible for combining and weighting features from previous layers to generate the final output. This structure enables neural networks to learn complex relationships and patterns within input data, providing powerful expressive capabilities. However, fully connected layers have a large number of parameters, leading to high computational costs. Particularly when dealing with large-scale data and deep networks, this may result in overfitting and excessive consumption of computational resources. Therefore, in modern neural network design, fully connected layers are often used in conjunction with other types of layers, such as convolutional and pooling layers, to improve model efficiency and generalization abilities.

The advantages of CNNs lie in their strong ability to learn representations from large-scale image data and their excellent performance in handling structured data such as images, effectively preserving spatial structure information through convolution operations. However, its disadvantages include the need for extensive training data and computational resources. In industrial and scientific algorithm research, there is often a shortage of sample data, necessitating the use of data augmentation techniques to improve performance. With the development of CNNs, an increasing number of classic algorithm models have emerged, corresponding to higher demands for hardware and devices. In summary, CNNs, as powerful deep learning models, have achieved tremendous success in the field of image processing and analysis, demonstrating outstanding performance and flexibility in practical applications. With technological advances and further research, CNNs will continue to play a crucial role in multiple domains.

### 3.2. Faster R-CNN

**Figure 4:**
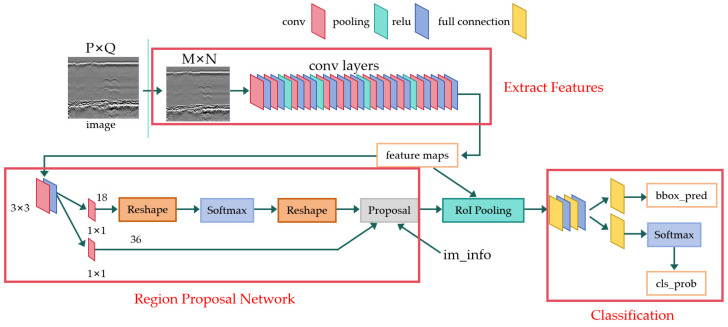
Architecture of Faster R-CNN.

Faster R-CNN is an object detection algorithm trained end-to-end, proposed by Ross Girshick [35] in 2015. The basic architecture of Faster R-CNN is illustrated in Figure 4. Its main features include using a region proposal network (RPN) to generate candidate regions and employing a single network to perform both region proposal and detection simultaneously. 

The structure of Faster R-CNN comprises four main components: the convolutional base network, the RPN module, region of interest (RoI) pooling, and the object classification and regression module. Firstly, the convolutional base network (such as ResNet or VGG) maps the input image to feature maps through feature extraction layers. Next, the RPN module slides over these feature maps to extract candidate regions (proposals or bounding boxes) and predicts whether each window contains an object and its bounding box coordinates. Subsequently, RoI pooling maps these candidate boxes onto the feature maps and adjusts them into fixed-size feature vectors. Finally, these feature vectors are fed into the object classifier and bounding box regressor. The classifier categorizes the candidate boxes into different object classes, while the regressor refines the bounding box positions to more accurately encapsulate the objects, achieving precise object classification and bounding box regression. The algorithm’s strength lies in its end-to-end training approach, enabling Faster R-CNN to achieve excellent performance in object detection tasks with generally superior prediction results compared to other algorithms. Additionally, Faster R-CNN can adapt to different application scenarios and requirements by integrating with various convolutional base networks. This algorithm has become a significant milestone in the field of object detection. 

### 3.3. YOLO

YOLO was originally proposed by Joseph Redmon et al. [36] in 2016, and subsequent versions YOLOv2 [53] and YOLOv3 [54] also originated from Joseph Redmon. YOLOv4 was introduced by Alexey Bochkovskiy et al. [55] in 2020, while YOLOv5 was proposed by the Ultralytics team in 2020. YOLOv7 and YOLOv9 were introduced by Chien-Yao Wang et al. [56,57]. The hallmark of this series of algorithms is their speed, making them capable of real-time object detection.

**Figure 5:**
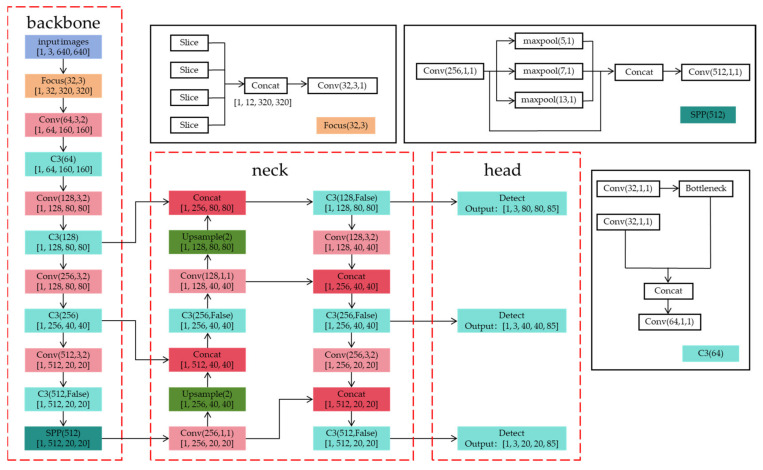
Architecture of YOLOv5s.

YOLOv1 is the first version of the YOLO algorithm, which treats object detection as a regression problem. It directly predicts bounding boxes and class probabilities using a single convolutional neural network. YOLOv1 divides the input image into a grid and predicts bounding boxes and class probabilities for each grid cell. Its main feature is fast inference speed, but it has limitations in detecting and localizing small objects. Building upon YOLOv1, YOLOv2 introduces improvements such as batch normalization, a high-resolution classifier, and anchor boxes. Additionally, YOLOv2 introduces joint training, allowing for the detection of classes from multiple datasets within a single model, enabling the detection of both common and custom objects. YOLOv3 introduces several enhancements, including a larger network, multi-scale feature maps, and a feature pyramid network (FPN) [58], achieving a better balance between speed and accuracy and improving small object detection performance. YOLOv4 further enhances detection accuracy and speed by incorporating the cross-stage partial darknet 53 (CSPDarknet53) as the backbone network, using techniques like the mish activation function and cross-stage partial connections. In contrast to previous versions, YOLOv5 is implemented in PyTorch 1.4 with a lighter network structure and improvements such as automatic data augmentation and multi-scale training to enhance detection performance and efficiency. The architecture of YOLOv5s is illustrated in Figure 5. YOLOv7 introduces various trainable Bag of Feature (BoF) modules to enhance detection without additional inference costs, further improving detection speed and accuracy. YOLOv9 introduces the concept of programmable gradient information (PGI) to obtain reliable gradient information for updating network weights. It designs a new lightweight network architecture, a generalized efficient layer aggregation network (GELAN), demonstrating the effective use of PGI on lightweight models. 

Overall, the YOLO series is characterized by its simple and direct design, fast inference speed, and suitability for applications requiring high-speed performance.

### 3.4. SSD

SSD is a single-stage object detection algorithm proposed by Wei Liu et al. in 2016 [37]. This algorithm utilizes CNNs as its backbone network and performs object detection on feature maps at different scales to accommodate objects of varying sizes. Compared to traditional two-stage detectors, SSD directly extracts features from images and predicts the categories and positions of objects, thereby achieving higher speed and good accuracy. Its multi-scale detection capability enables effective detection of objects of various sizes, making it widely applicable in real-time object detection and industrial applications. By introducing a multi-task loss function, SSD simultaneously optimizes object classification and bounding box regression, further enhancing model performance. These features have led to extensive adoption of SSD in real-time object detection and industrial applications, marking significant progress in the field of object detection. In comparison to other single-stage algorithms, SSD stands out for its simple and efficient design, maintaining high detection accuracy while achieving faster inference speeds. Even with smaller input image sizes, SSD demonstrates superior performance. 

### 3.5. U-Net

**Figure 6:**
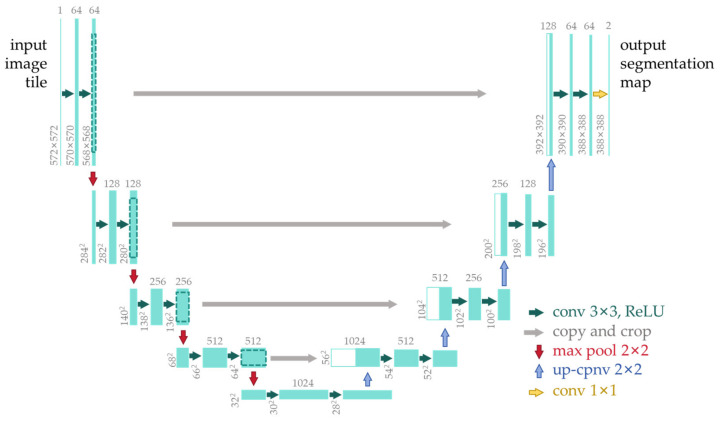
Architecture of U-Net. Reprinted with permission from ref. [38].

U-Net is a convolutional neural network designed for biomedical image segmentation, initially proposed by Ronneberger et al. in 2015 [38]. The basic architecture of U-Net is illustrated in Figure 6. The network structure consists of symmetric encoder and decoder components: the encoder reduces the size of the input image through convolution and pooling operations, extracting high-level abstract features, while the decoder upsamples the feature maps to the original size, generating pixel-level segmentation results. One of U-Net’s distinctive features is skip connections, which link corresponding layers between the encoder and decoder. This facilitates the transmission of fine-grained information and helps prevent information loss, thereby enhancing segmentation accuracy. Additionally, U-Net often incorporates data augmentation techniques such as random rotations, flips, and scaling to increase the diversity of training data and improve model generalization. Originally designed for biomedical image segmentation, U-Net’s performance and simplicity have led to its wide application in other fields, such as satellite image segmentation and road recognition. In practical applications, U-Net typically performs well, particularly in medical image segmentation tasks, achieving satisfactory results and becoming one of the preferred models in many real-world applications.

In summary, Faster R-CNN, YOLO series, SSD, and U-Net are commonly used algorithms in the field of object detection, each with distinct characteristics and advantages. For instance, Faster R-CNN excels in high-precision object detection, YOLO and SSD are more suitable for real-time detection requirements, and U-Net is often utilized for image segmentation tasks. 

For different NDT methods, various factors also need to be considered when choosing the appropriate algorithm. Each method produces different signal characteristics, such as images, waveforms, spectra, or other types of data. When choosing algorithms, it is essential to select those capable of processing and analyzing these specific data types. For example, waveform data obtained from PAUT requires preprocessing like denoising and signal enhancement before transforming it into a format recognizable by the algorithm for training. Jayasudha et al. [59] utilized adaptive least mean square (ALMS) [60] to enhance PAUT ultrasonic signals to eliminate noise from detection signals. They applied empirical wavelet transform (EWT) [61] to convert time-domain signals into frequency-domain signals, followed by the classification of PAUT welding signals for defect detection using a deep convolutional neural network (DCNN) [62]. In scenarios requiring real-time monitoring and automated analysis, such as data analysis in PAUT and AE, faster and more efficient algorithms are typically necessary. Siddique et al. [63] proposed a pipeline leakage diagnosis framework based on CNNs, convolutional autoencoders (CAEs) [64] and an artificial neural network (ANN) [65] to identify AE scalogram images obtained from continuous wavelet transform. This framework first extracts signal features using a CNNs and a CAE, followed by using ANN to assess the pipeline’s health condition.

## 4. Data Augmentation Methods

Deep learning models require large annotated datasets for training to prevent overfitting. However, common forms of data in NDT methods often include signals, peaks, textures, and other types that are challenging to statistically analyze, collect, and process. Implementing various NDT methods also consumes significant time and resources. Therefore, acquiring a sufficient amount of appropriate data for NDT poses a significant challenge. To address this, employing data augmentation methods becomes necessary to expand datasets, providing essential support for in-depth research into various NDT techniques. 

### 4.1. Traditional Data Augmentation Methods

**Figure 7:**
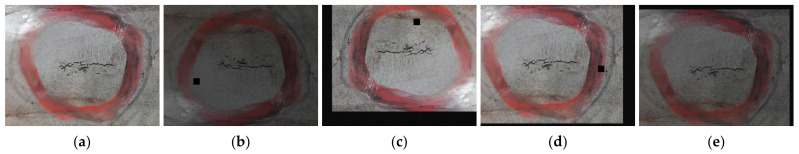
Sample data before and after traditional data augmentation methods: (**a**) Original picture; (**b**) Random flip + Cutout + Adjust brightness; (**c**) Random translation + Random rotation + Cutout; (**d**) Random translation + Cutout; (**e**) Random translation + Add noise + Adjust brightness.

The traditional data augmentation methods are based on geometric and pixel transformations of existing training samples. These include random translation, cropping, rotation, flipping, noise addition, cutout, and brightness adjustment operations. Among them, as shown in Figure 7, traditional data augmentation methods for crack defect detection in magnetic particle testing technology are applied. For most NDT data samples of pressure-bearing equipment, geometric transformations are the most commonly used data augmentation techniques, while pixel transformation operations are less frequently employed due to their potential to alter fundamental data properties. Traditional data augmentation methods can enhance the accuracy of network training within a certain range. However, they may encounter training saturation issues [66], where further improvements in training effectiveness cannot be achieved. This is primarily because traditional methods do not introduce any new additional information required for the data [52].

### 4.2. Data Augmentation Methods Based on Deep Learning

**Figure 8:**
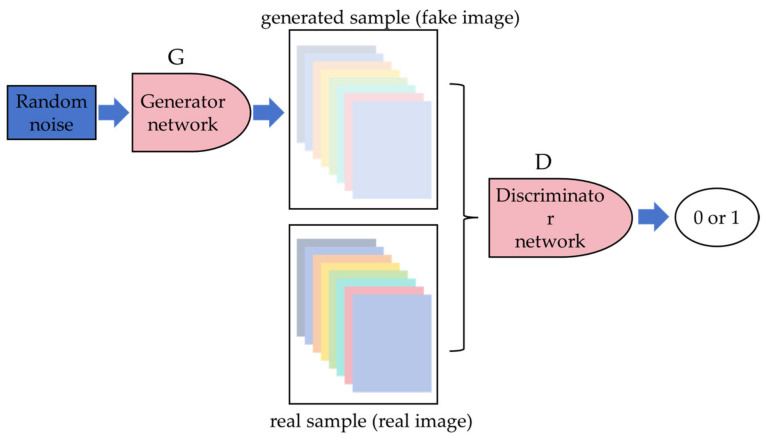
The basic architecture of the GAN.

The Generative Adversarial Network (GAN) is a deep learning model proposed by Canadian computer scientist Goodfellow [67] in 2014. The GAN consists of two main components: the generator and the discriminator. Figure 8 illustrates the basic architecture of the GAN. The generator takes random noise or latent space vectors as input and maps them to a data space similar to the training data. Its goal is to generate samples that appear realistic enough to deceive the discriminator. The discriminator, on the other hand, distinguishes between fake samples generated by the generator and real training data. It takes samples as input and outputs a probability indicating the likelihood that the input sample is real data.

During training, the generator and discriminator engage in an adversarial process: the generator strives to produce increasingly realistic samples to fool the discriminator, while the discriminator aims to accurately differentiate between real and fake samples. Through this adversarial training process, both the generator and discriminator gradually enhance their performance, ultimately reaching a dynamic equilibrium where the generator produces samples realistic enough that the discriminator cannot reliably distinguish between real and fake.

In recent years, with further advancements in GAN technology and the emergence of related network models, GAN has achieved significant success and applications in areas such as image generation, style transfer, super-resolution image reconstruction, and data augmentation. However, studies indicate that the training process of GANs can be relatively unstable, resulting in potentially distorted generated images. The careful adjustment of hyperparameters and network architecture is required. Utilizing GAN-generated samples for expanding datasets can help reduce model overfitting and improve the accuracy and generalization capability of object detection algorithms in scenarios with limited sample sizes [52].

Jain et al. [68] separately trained three different GAN architectures—deep convolutional GAN (DCGAN) [69], auxiliary classifier GAN (ACGAN) [70], and Information-theoretic GAN (InfoGAN) [71]—to generate enhanced image samples for evaluation of model performance. Subsequently, they trained a CNNs classification model and compared GAN-based enhancement methods with traditional augmentation techniques. Comparative experimental results showed that the sensitivity of the CNNs enhanced with DCGAN-based data augmentation was 95.33%, with a specificity of 99.16%, significantly outperforming a traditional CNNs with standard augmentation methods, resulting in a more effective and robust model. Posilović et al. [72] compared three methods for generating defective ultrasound images based on real data: traditional copy–paste method [73], the detectionGAN [74] model, and a spatially adaptive denormalization (SPADE GAN) [75] model. They evaluated these methods through expert human assessment, confirming that images generated by deep learning generative networks cannot be distinguished from real ultrasound images. Additionally, their previous work introduced a detectionGAN model to generate ultrasound images with defects in different locations, which improved the performance of deep convolutional neural networks. Specifically, the average accuracy of defect detection performance trained on real data was 71%, increasing to 72.1% when trained solely on generated data. By combining generated and real data, they achieved an average accuracy of 75.7%.

Guo et al. [76] proposed a contrast enhancement conditional GAN (CECGAN) model based on the radiographic image data imbalance problem. The experimental results demonstrate that this defect detection model can identify five types of welding defects: cracks, lack of fusion, lack of penetration, pores, and slag inclusions, achieving an F1 score of 0.909 and a defect recognition accuracy of 92.5%. Jiang et al. [77] introduced a GAN-based TOFD image reconstruction model and a conventional weld seam recognition method. They first designed the image-wave feature fusion (IWFF) module based on depth separable convolution to integrate and analyze image and wave features, establishing the IWFF-GAN model. The results show that this approach accurately distinguishes between normal and abnormal weld seams, achieving a high accuracy in normal weld seam recognition. The area under the receiver operating characteristic curve is 0.9903. Ren et al. [78] addressed the issue of missing samples in MFL detection by proposing a method that combines conditional autoencoder (CVAE) and GAN to reconstruct data and handle MFL sample deficiencies effectively, thereby generating a large number of various defect samples. The effectiveness of this method is demonstrated. Table 1 summarizes existing applications of GAN-based data augmentation in common NDT techniques for pressure equipment.

Due to the typically small-scale datasets in industrial applications and scientific research, data augmentation techniques based on deep learning demonstrate significant potential and advantages for industrial use. They can greatly reduce the time and opportunity costs involved in collecting sample data. Applying GAN technology to enhance signal and image data related to NDT represents an important and inevitable trend in development. 

## 5. Application of Object Detection Algorithms in Pressure Equipment NDT

The NDT techniques described above have been partially applied to defect identification of pressure equipment based on the algorithmic model of object detection, achieving excellent results. Although mostly in the experimental stage, it is undeniable that the development and application of this combined technology represent an inevitable trend for the future application of NDT techniques in industry and other fields. The introduction of artificial intelligence provides a new and efficient solution for the identification of abnormal signals in NDT. This section will focus on the application of deep learning-based target detection algorithms in NDT techniques.

Combining target detection algorithms with NDT techniques for automatically identifying common defects in inspection signals is a promising approach, but it also faces several challenges. Target detection algorithms are typically used to detect and locate specific objects or features from images or videos, such as object detection or facial recognition. When applying target detection to analyze NDT signals, several considerations must be taken into account:

Signal preprocessing and feature extraction: Most NDT signals have high sampling frequencies and contain significant information, making them more complex to process. Before applying target detection algorithms, preprocessing and feature extraction of the inspection signals are often necessary to enable effective processing and analysis by the algorithms. Techniques like filtering, enhancement algorithms, etc., can be used to denoise and remove interference, ensuring more accurate and visualized detection results.

Data annotation and training: Target detection algorithms typically rely on large amounts of annotated data for training to learn and recognize different categories of targets. Since most NDT signals are non-intuitive waveforms or challenging to distinguish image signals, the data annotation process can be more complex and time-consuming. This is because it requires specialized personnel to annotate different types of defect features. Ensuring the quality and accuracy of the labeled data is critical to the performance of the algorithm, which can add to the cost and complexity of implementing the method, which is a significant challenge when implementing the method.

Diversity of defect types: NDT may involve various types of defects such as cracks, pores, inclusions, etc. Target detection algorithms need to identify and distinguish these different types of defects and handle targets of different scales and shapes. This requires algorithms with sufficient classification and localization accuracy and robustness to handle complex detection scenarios, ensuring accurate assessment of equipment reliability and safety.

Real-time performance and efficiency of algorithms: In practical applications, NDT of pressure equipment often requires fast response times and high efficiency. Target detection algorithms need to process inspection signals in real-time or near real-time conditions, maintaining stability and reliability in complex working environments. This necessitates optimizing computational performance, algorithm complexity, and efficient utilization of hardware resources during the algorithm design phase.

Despite the challenges involved, there remains significant potential in combining object detection algorithms with NDT techniques. With advancements in deep learning technology and hardware capabilities, handling complex detection signals has become more efficient and stable. Overall, the success of this approach hinges on effectively integrating and optimizing detection signals, object detection algorithms, and engineering requirements in practical applications. Addressing the technical and engineering challenges requires interdisciplinary collaboration and in-depth technological research. 

### 5.1. Application of Object Detection Algorithms in MT

As one of the most commonly used NDT methods in ferromagnetic material inspection, MT technology has been widely used in the field of pressure equipment inspection as it can visualize the shape, location, and size of the defects [79,80,81]. It is primarily used for detecting surface and near-surface defects. The basic principle relies on the interaction between a magnetic field and ferromagnetic materials. Iron particles are sprinkled on the surface of the material being inspected. When a magnetic field is applied, areas of magnetic flux leakage form around cracks or defects, attracting the iron particles and creating visible lines or spots. This method aids in determining the position and shape of defects, enabling sensitive detection of surface flaws. 

MT technology, as an NDT method, excels in detecting surface cracks, corrosion, and other defects. However, traditional MT heavily relies on manual observation and experiential judgment, which may pose limitations in tasks involving extensive inspections, complex environments, and challenging conditions such as heights, dust, and strong winds. To address these challenges, integrating object detection techniques into MT can facilitate faster and more accurate defect identification. Moreover, the distinctive feature of this NDT method lies in its use of magnetic particle imaging, which provides visible and discernible information for human judgment. The captured image data are conducive to meeting the basic requirements of object detection algorithms. Thus, these conditions offer feasibility for the integration of object detection algorithms into magnetic particle testing. 

Currently, there are few research cases integrating object detection algorithms with MT in the field of pressure equipment inspection. Shunsuke et al. [82], aiming to automate MT detection, employed an enhanced U-Net algorithm to discern the presence or absence of MT defects. The model achieved an average recognition accuracy of 85.8%. In the future, this detection method could be integrated into unmanned aerial vehicles or inspection robots. For instance, drones equipped with magnetic particle testing instruments and object detection algorithm modules could inspect surface defects on large-scale pressure equipment. This could replace manual inspections in harsh environments, such as heights, climbing, strong winds, and dust, thereby driving innovation and development in the field of pressure equipment inspection and testing.

### 5.2. Application of Object Detection Algorithms in PT

PT technology, based on liquid penetration and imaging principles, is suitable for detecting surface and near-surface cracks, holes, and other surface defects in materials. Its workflow involves coating the surface under inspection with a high-surface-tension penetrant, allowing it to penetrate into defects, and then using a contrasting developer to reveal these defects. This method is simple to operate, cost-effective, and applicable not only to metals but also to plastics, ceramics, and other materials. It is equally effective for objects with complex structures and shapes. Due to its non-destructive, rapid, and precise nature, penetration testing finds extensive application in industries with high precision and reliability requirements, such as aerospace, manufacturing, and pressure equipment inspection. It helps ensure product quality and production safety. 

PT is able to effectively detect defects such as tiny cracks and porosity on metal surfaces by coating penetrant and developer but it usually requires manual observation and evaluation. The introduction of target detection algorithms, on the other hand, enables automated defect identification and localization, which greatly saves manual operation time and improves the real-time efficiency of inspection.

Currently, there is partial research on the combination of PT technology with target detection algorithms in the aerospace industry [83,84,85]. Although there are few research cases in pressure equipment inspection, this combined technology exhibits strong applicability for detecting surface defects in various inspection objects, showing potential for research and application in pressure equipment inspection. Niccolai et al. [84] developed a vision expert system based on deep learning for automated detection of fluorescent penetrant inspection (FPI) in aerospace manufacturing, leveraging machine learning to enhance detection accuracy. Shipway et al. [85] explored the potential of using deep learning methods for automated FPI detection, demonstrating that ResNet34 and ResNet50 architectures significantly improve defect detection accuracy compared to traditional Random Forest methods under the conditions of small datasets.

### 5.3. Application of Object Detection Algorithms in RT

**Figure 9:**
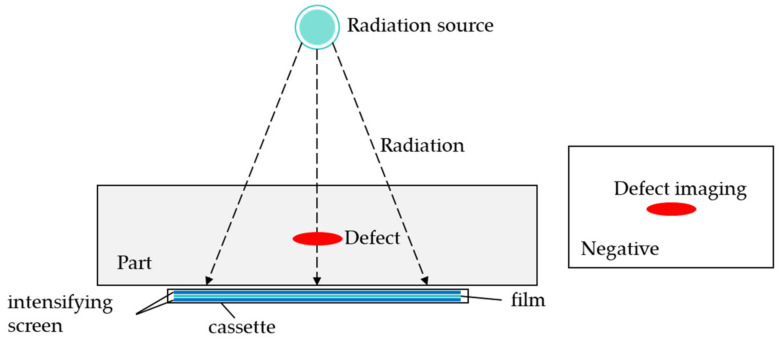
Schematic diagram of RT principle.

RT is a technique that utilizes X-rays or gamma rays to penetrate materials and measure radiation absorption for detecting internal defects [86,87,88]. Depending on the density and thickness of the tested component, incident X-ray photons undergo varying degrees of attenuation during transmission and also scatter due to interactions with atoms. If a component has a defect or variation in material properties or thickness, incoming X-ray photons will be attenuated differently as they pass through. Subsequently, the transmitted X-ray photons create a latent image, which is captured using X-ray film or digital detectors and then converted into a two-dimensional radiographic image of the component. Therefore, radiographic images in NDT provide crucial information about the internal structure of objects and can reveal internal defects. The schematic diagram of the RT principle is shown in Figure 9.

In the inspection of special equipment, RT is widely used for detecting pressure-bearing devices, such as metal materials, pressure vessels, and pipelines, commonly found in industries like manufacturing, chemical, petroleum, and natural gas. RT involves the use of X-rays or gamma rays to penetrate the object being inspected, producing images that can be analyzed to determine defects such as cracks, lack of fusion, lack of penetration, porosity, and slag in the inspected areas. However, this determination process typically requires experienced engineers or experts to identify and analyze these defects. In industrial RT tasks, the workload is often substantial, and the identification process is complex, leading to high labor costs and low efficiency. To address this challenge, many scholars, both domestically and internationally, have explored precise identification and determination of defects in radiographic images by training and improving object detection algorithm models [4,5,89,90,91]. 

#### 5.3.1. X-ray

Currently, deep learning-based object detection algorithms are being gradually applied to the identification of defects and anomalies in X-ray images. Hena et al. [4] explored the effects of signal-to-noise ratio (SNR) and contrast-to-noise ratio (CNR) on the performance of the U-net deep learning semantic segmentation model for automatic X-ray image defect recognition using deep learning. The results show that the model with high CNR values yielded an intersection-over-union (IoU) [92] metric of 0.9594 on test data of the same category but dropped to 0.5875 when tested on lower CNR test data. Zhang et al. [5] proposed the automated anti-counterfeiting detection of X-ray welded film and extracted weld characteristics based on an improved SPP-net deep learning model. The results show that the proposed method can accurately locate and segment welding seam, extract welding seam features, characters and markers information of the welding films, and automatically complete forgery detection based on welding seam fingerprint matching and overlap matching. Ji et al. [89] proposed a deep learning-based defect detection model using the two-stage detection network Faster R-CNN as the foundational model. Their approach integrated the FPN and the squeeze and position attention mechanism (SPAM) to detect X-ray defects of various shapes and locations. They achieved a precision with mean average precision (mAP) [93] of 86.3%.

#### 5.3.2. Digital Radiography (DR)

Possibly due to its comparative disadvantage in applicability and scope compared to other NDT techniques, DR appears somewhat limited. Currently, there are few research cases on defect anomaly recognition in DR images using deep learning-based object detection algorithms. However, it is undeniable that the application of this combined technology exhibits strong compatibility, substantial potential, and superior conditions.

Wang et al. [90] developed a CNN-based model enhanced with a self-attention guidance module (SGM) to achieve precise detection of casting defects in DR images. Their system achieved an accuracy of 91.17% in identifying 20 different types of defects and non-defective cases. Hai et al. [91] employed the gain-adaptive multi-scale retinex (GAMSR) algorithm and combined it with the FPN and the convolutional block attention module (CBAM) [94] to address the detection of minute porosity defects. Their experimental results significantly enhanced the detection performance of DR images of aluminum castings, surpassing the mAP values obtained using classical networks like YOLOv5, YOLOv7, and Faster R-CNN.

RT technology penetrates deeply into the internal structures of equipment with its high-resolution imaging capability, effectively detecting minute defects, such as cracks and voids. However, the analysis and interpretation of its data typically rely on experienced technical personnel. With the introduction of object detection algorithms, these common defects can be automatically identified and located, significantly enhancing detection accuracy and efficiency while reducing subjective judgment and errors inherent in human assessment.

### 5.4. Application of Object Detection Algorithms in Ultrasonic Inspection

Similarly, traditional ultrasonic inspection techniques, when applied to pressure-bearing equipment, still rely on experienced engineers or specialists for the identification and analysis of defect signals corresponding to various types of defects. However, unlike X-ray inspection, ultrasonic signals detected using ultrasonic inspection are less intuitive, requiring higher technical expertise and experience from inspection personnel. Given these challenges, there appears to be a growing need for an intelligent and efficient method for anomaly detection in ultrasonic signals. Object detection algorithms hold unique advantages in this field. Currently, a considerable number of researchers have studied anomaly detection of ultrasonic signals under different defect conditions using various object detection models, achieving high levels of accuracy.

#### 5.4.1. UT

UT is one of the primary NDT methods for pressure equipment. Its principle involves the reflection of ultrasonic waveforms from materials and defects and determining the location and nature of defects, such as cracks, inclusions, and voids, based on changes in penetration time [6,95,96,97]. UT offers the advantages of high sensitivity and quantitative analysis capabilities. The disadvantage is that the defects cannot be displayed intuitively, and the surface of the parts to be tested requires smoothness. In addition, ultrasonic transmission requires a coupling agent due to the high consumption between medium and air.

Kim et al. [11] developed a multi-branch deep fusion network (MBDFN) deep learning algorithm model for identifying and classifying four types of weld seam defects from ultrasonic signals. The algorithm achieved an accuracy of 92.2% in defect recognition and classification. The study also discussed the prospective application of this deep learning model in automated welding robots or welding inspection systems. Medak et al. [98] designed a deep learning architecture named DefectDet to detect defects in UT images. Compared to their previous research [99] using EfficientDet-D0 [100] for ultrasonic defect recognition, the model showed an improvement in accuracy by 1.7% (512 × 512 px input resolution) and 2.7% (384 × 384 px input resolution), with an mAP reaching 0.913.

#### 5.4.2. TOFD

TOFD is a high-precision NDT technique that utilizes the principles of ultrasonic diffraction and time-of-flight measurement [101,102]. It employs longitudinal wave transducers to generate wide-beam ultrasonic waves and simultaneously captures diffraction signals reflected from defects. These diffraction signals appear as distinct bright spots or peaks in TOFD images, indicating the location and depth of defects. Due to its high spatial resolution and accurate defect sizing capabilities, TOFD finds extensive applications in NDT of engineering structures. Zhi et al. [103] proposed a titanium alloy welding defect recognition method based on Faster R-CNN, termed the enlighten Faster region-based convolutional neural network (EFRCNN). This method achieves localization and recognition of five types of defects in TOFD images: porosity, cracks, slag inclusion, lack of penetration, and lack of fusion. Comparative evaluations with several traditional Faster R-CNN architectures demonstrate that EFRCNN exhibits superior accuracy, achieving a defect recognition accuracy of 92.35 ± 2.04%.

#### 5.4.3. PAUT

PAUT utilizes multi-element array probes to achieve multiple-angle ultrasonic beam emission and dynamic focal spot control by precisely controlling the excitation time and amplitude of each element [3,104,105]. This technology enables coverage of a wide inspection area without moving the probe while adjusting the focal spot position in real time to enhance detection sensitivity and accuracy. PAUT generates high-resolution internal structure images through real-time imaging and data processing, facilitating high-precision detection of internal defects and structures within the target object.

Jayasudha et al. [59] employed adaptive least mean square (ALMS) [60] to enhance PAUT ultrasonic signals for noise elimination in defect detection. They utilized empirical wavelet transform (EWT) [61] to convert time-domain signals into frequency-domain signals. Finally, they used a DCNN [62] to classify PAUT welding signals for defect presence, achieving a precision of 97%. Chen et al. [106] proposed an improved YOLOv8_SBA network architecture to enhance the accuracy and efficiency of PAUT defect detection. They introduced space-to-depth convolution (SPD-Conv) [107] to replace strided convolution, constructed a Bi-level routing and spatial attention (BRSA) module integrated into the backbone, and adopted asymptotic feature pyramid network (AFPN) [108] in place of the original structure. This approach improved the identification and determination of flat bottom holes (FBH), side-drilled holes (SDH), and defect-free regions, enhancing the accuracy, precision, and robustness of ultrasonic defect detection. Sudharsan et al. [109] developed a multimodal automatic defect recognition (M-ADR) system comprising Tri-planar mask R-CNN (T mR-CNN) and the Bi-planar medial axis transform (Bi-MAT) algorithm. By integrating PAUT with pulsed thermography (PT) technology, they achieved precise identification of defect features and determination of defect sizes. The Bi-MAT algorithm could measure SDH defects as small as 0.3 mm and cracks up to 18 mm in size. T mR-CNN achieved a probe accuracy of 91.46%, with F1 scores reaching 61.62%. 

Ultrasonic inspection technology, as an important NDT method, features high resolution, deep penetration, and non-invasiveness, enabling effective detection of minute defects and structural changes within materials. The integration of target detection algorithms with ultrasonic inspection not only promises breakthroughs in real time and precision aspects of NDT in pressure equipment but also holds potential for broader applications in complex scenarios and industries such as aerospace, automotive manufacturing, and nuclear power.

### 5.5. Application of Object Detection Algorithms in MFL

**Figure 10:**
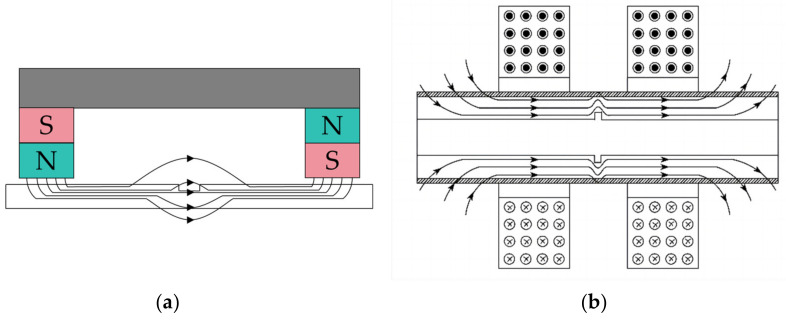
Basic principle of MFL testing: (**a**) yoke-type magnetizer; (**b**) encircling coil-type magnetizer. Adopted from [114].

MFL technology is based on the phenomenon of magnetic induction and the magnetic properties of objects [110,111,112,113]. In industrial applications, MFL is typically used to detect magnetic defects or anomalies on the surface or inside metallic components. This technology utilizes electromagnetic induction by introducing a magnetic field around or within the object under examination, and then measuring its response to this magnetic field. When there are magnetic irregularities, cracks, or other defects present on the surface or inside the object being inspected, these abnormal areas exhibit different effects on the applied magnetic field, such as distortion or reflection. Detectors or sensors analyze these feedback signals to identify and locate the presence and nature of defects. MFL technology holds significant importance in the quality control and inspection of components such as aerospace engine blades, automotive engine parts, pipelines, and containers [113,114,115,116]. The basic principle of MFL testing is schematically shown in Figure 10.

The MFL technique detects surface and subsurface defects in pipelines or containers by sensing variations in the magnetic field on their walls. Its detection signals are characterized by high sensitivity and spatial resolution. Object detection algorithms excel at extracting key features from complex MFL signals, enabling effective identification, classification, and quantification of different types of defects. Yuksel et al. [117] proposed a quantitative method for MFL defect detection based on swin transformer backbone YOLOv5 (SwinYv5) and cross-residual convolutional neural network (CR-CNN), which interprets MFL signals obtained using a semi-automatic online inspection robot through the use of MFL sensors. The method achieved high-precision defect detection with maximum errors of 1.30 mm, 1.65 mm, and 0.47 mm for length, width, and depth defect detections, respectively. Zhang et al. [118] proposed a VDTL neural network that not only predicts the sizes of MFL defects in oil and gas pipelines but also estimates the cross-sectional profiles of the defects.

### 5.6. Application of Object Detection Algorithms in ECT

**Figure 11:**
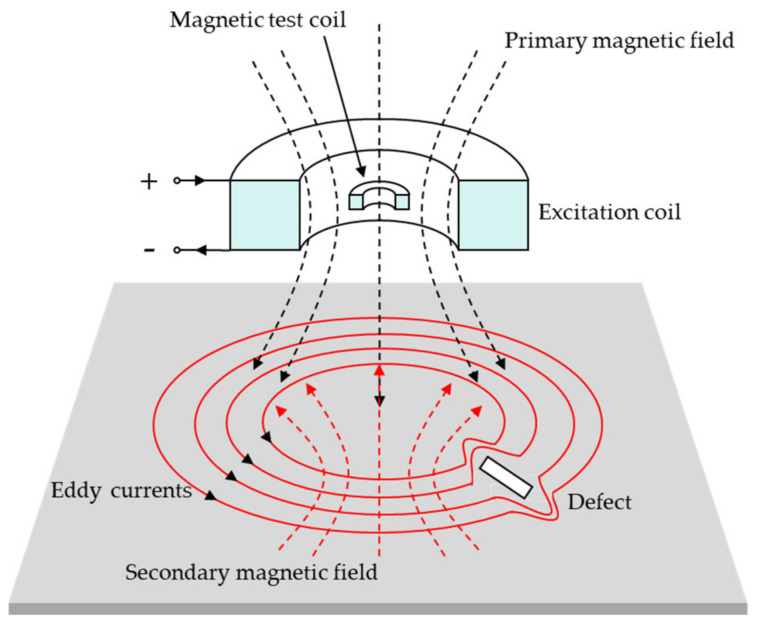
Schematic diagram of the ECT principle.

The basic principle of ECT is the use of electromagnetic induction to detect defects in conductive materials [3,119,120,121]. An alternating current is applied to the ECT inspection coil, which thus generates an alternating magnetic field perpendicular to the workpiece due to Faraday’s law of electromagnetic induction. When the inspection coils are in close proximity to the workpiece being examined, eddy currents are generated on the outer surface of the workpiece. These eddy currents produce a magnetic field that opposes the original magnetic field, resulting in a change in the resistance and inductance of the inspection coils. If there is a defect in the workpiece to be tested, it will change the magnitude of the eddy currents and the strength of the induced magnetic field, which will lead to a change in the coil resistance, and eventually, the changes can be detected to determine the defects or not [6]. The principle schematic diagram of ECT is shown in Figure 11.

The ECT technique is characterized by its high sensitivity, rapid response, and the advantage of non-contact testing of the surface of objects. However, ECT images are often subject to noise interference from various sources such as electromagnetic interference and environmental background variations. These factors pose challenges for the accurate detection of weld defects. Object detection algorithms offer effective solutions to these issues. By training deep neural networks to recognize and locate various defects within weld seams, precise localization and classification can be achieved in complex ECT images. Tao et al. [122] proposed a novel differential multimodal flexible ECT probe for weld seam inspection. To suppress image noise, they proposed a spatial domain filtering and gradient feature edge extraction (SDF-GFEE) image preprocessing algorithm and employed mask region convolution neutral network (Mask R-CNN) for detecting weld seam defects in ECT images. They achieved detection of groove defects measuring 3 mm (length) × 0.1 mm (width) × 0.5 mm (depth) and FBH sized at Φ 0.8 mm × 0.5 mm. Meng et al. [123] developed a highly integrated portable ECT device and utilized it to construct a dataset. They subsequently trained and evaluated several state-of-the-art one-dimensional residual CNNs on this dataset. The results demonstrated that the 38-layer network ResNeXt1D-38 achieved a precision of 93.58% in detecting surface defects ranging in depth from 0.3 to 2.0 mm at a resolution of 0.1 mm.

### 5.7. Application of Object Detection Algorithms in AE

AE technique identifies and assesses the formation and propagation of micro-cracks, defects, or damages in materials or structures during loading processes by monitoring high-frequency acoustic waves emitted [124,125,126]. It utilizes highly sensitive AE sensors to capture minute acoustic signals and provides critical information about the structural health status through analysis of signal amplitude, frequency, and duration characteristics. AE technology is characterized by its non-destructive nature, high sensitivity, real-time monitoring, and strong localization capabilities, making it widely applicable in fields such as aerospace, construction, and pressure equipment inspection and monitoring. It plays a crucial role in ensuring structural integrity and safety.

Currently, deep learning algorithms combined with AE technology have been partially researched in damage monitoring [127,128,129] and damage identification [130,131] of composite materials, railway tracks [132,133,134,135], and defect recognition in pressure equipment. AE technology detects defects by monitoring small stress-release events within equipment. However, traditional AE methods suffer from low recognition accuracy and significant false positives. Introducing object detection algorithms effectively addresses these shortcomings by employing deep learning algorithms for precise analysis and pattern recognition of signal data, thereby enhancing the capability to identify structural defects in equipment. Siddique et al. [63] proposed a pipeline leakage diagnosis framework using CNNs, convolutional autoencoders (CAE) [64], and artificial neural networks (ANN) [65] to identify AE scalogram images obtained from continuous wavelet transform. This framework first extracts signal features using a CNNs and a CAE, followed by an ANN evaluation of pipeline health, enabling the recognition of AE signals under different conditions and leakage sizes. Islam et al. [136] proposed a new method for crack identification in pressure vessels, which includes crack feature calculation, GA-based feature selection, and a deep neural network (DNN) for crack classification in AE testing. Experimental results demonstrate the method’s effectiveness in AE signal feature discrimination.

Table 2 summarizes the application of object detection algorithms in anomaly recognition of defect signals in NDT. Additionally, in many cases, the performance of manual inspection has not been effectively quantified and lacks sufficient reliability for direct comparison with the performance of object detection algorithms. Only a few studies have reported that performance metrics for anomaly detection and classification based on object detection algorithms, as measured by the probability of detection (POD), are sufficient to achieve human-level performance [73]. However, most results do not provide direct data or discussions comparing the detection performance of NDT technologies based on object detection algorithms with that of human experts but instead focus on performance comparisons between different algorithms and network models [89,90,91,103]. Nonetheless, from the perspective of technological improvement and the efficiency and auxiliary functions provided by object detection algorithms, integrating these algorithms with NDT technologies has undoubtedly significantly advanced the field of NDT. 

Despite the numerous advantages of combining object detection algorithms with NDT technologies, several challenges and limitations remain. First, object detection algorithms typically require a large amount of labeled data for training, and the data labeling work for NDT images is complex and time-consuming. Therefore, acquiring high-quality training and labeling data becomes a significant challenge. Second, the literature suggests that for some small targets with indistinct features, such as weak industrial defects, detection accuracy is insufficient and improvements to the algorithmic model are needed to enhance performance, such as incorporating attention mechanism modules [89]. Third, object detection algorithms, especially deep learning models, often have high computational complexity and require substantial computational resources, which may limit their effectiveness in real-time applications. Finally, the “black box” nature of deep learning models makes result interpretation more challenging, potentially reducing trust in the detection results. 

In the future, with the continued advancement of deep learning technology and the increase in hardware computational power, the combined application of object detection algorithms and NDT techniques is expected to achieve greater breakthroughs in terms of real-time performance, automation level, and data processing capability. This will enhance their application value and promote their wider adoption in industrial inspection and testing. In addition, the combined technique of NDT and target detection algorithms should undergo multidimensional data fusion analysis to comprehensively evaluate and analyze the nature, location, and size of defects. However, implementing such technological integration requires attention to data quality, algorithm selection and optimization, as well as system integration design to ensure the overall reliability and stability of the technology in practical applications. Therefore, the integration of NDT technology with target detection algorithms not only demonstrates the advantages brought by technological integration but also necessitates thorough research and optimization at various levels of technical and engineering implementation. 

## 6. Open Challenges

Although progress has been made in pressure equipment inspection and assessment based on target detection algorithms, several challenges remain. These primarily include the integration of semi-supervised, weakly supervised, and unsupervised learning, the combination of GAN data augmentation with unsupervised learning models, and interpretable model development. 

A significant amount of research in the field of target detection has focused on using annotated data for supervised learning. However, effectively utilizing semi-supervised, weakly supervised, or unsupervised learning techniques to improve the performance of target detection, especially in cases where annotated data are scarce or expensive, remains a critical challenge. Exploring how to combine these different learning paradigms to enhance the robustness and generalization capability of target detection algorithms is a key direction for future research.

Combining GAN data augmentation with unsupervised learning models represents a highly promising approach, particularly in scenarios where data are scarce or difficult to annotate. By leveraging GANs for data augmentation, additional data samples can be generated to expand the original dataset, thereby enhancing the model’s generalization capability and performance. This approach is especially applicable in fields rich in data such as images and videos. Simultaneously, unsupervised learning models emphasize learning useful features or representations from unlabeled data. By combining the capabilities of unsupervised learning with GANs, it becomes possible to effectively utilize information within the dataset without explicit labels to improve model performance. This method not only reduces the cost of manually labeling data but also addresses large-scale and complex datasets. Furthermore, the generator component of GANs can serve as a part of unsupervised learning models, aiding in learning the distribution characteristics and underlying structures of the data. This integration enhances the model’s performance on unlabeled data [69,137]. This combination not only addresses the issue of insufficient samples faced by most current target detection algorithms and research but also effectively tackles the high costs and human errors associated with annotating large datasets. This method optimizes the performance of existing tasks and the generalization capability of models, enhancing the development of relevant deep learning algorithms in the field of NDT. It provides more efficient and innovative solutions for various NDT technologies and applications.

Deep learning models are often considered difficult to interpret due to their complexity and black-box nature. However, several methods and techniques attempt to enhance their interpretability, allowing for clearer explanations of their decision-making processes and predictive outcomes. These models can provide explanations or reasons for their decisions, including feature importance analysis (FIA), local interpretable model-agnostic explanations (LIMEs) [138], SHAP values (SHapley Additive exPlanations) [139], and visualization tools (VTs). These approaches not only improve the transparency and credibility of the models but also help identify potential model biases or errors. 

## 7. Conclusions

Developing intelligent NDT technologies for pressure equipment helps to improve detection accuracy and efficiency, reduce the workload and human errors of inspectors, and promote high-quality development of inspection methods for pressure equipment. The application of object detection algorithms and deep learning technologies in the field of NDT for pressure equipment is of significant importance. These technologies can enhance detection accuracy, reducing the risks of missed detections and false alarms. Moreover, they enable rapid processing and analysis of large volumes of image or video data, thereby improving detection efficiency. Additionally, by identifying defects and automatically classifying NDT images or signals, high-precision and automated NDT can be achieved. Introducing technologies such as object detection algorithms and deep learning into NDT for pressure equipment can enhance equipment safety, reliability, and operational efficiency, thus playing a crucial role in the inspection and testing of pressure equipment in industrial applications.

In recent years, convolutional neural network-based deep learning algorithms have advanced rapidly. Therefore, it is foreseeable that the development of deep learning algorithms will inevitably drive progress in the field of NDT. Furthermore, with the advancement of smart detection fields and technologies for pressure equipment in the future, there will also be a demonstrated demand, both domestically and internationally, for unified standards and technical specifications regarding the inspection of pressure equipment using various intelligent detection technologies and methods.

## 8. Outlook

Future research can focus on further enhancing the detection performance and accuracy of algorithms. This can be achieved through optimizing network structures, adjusting hyperparameters, transforming detection signals, and introducing more advanced object detection algorithms. These efforts aim to meet various types and characteristics of NDT tasks, thereby improving detection accuracy and robustness. Additionally, adapting algorithms to different environments, lighting conditions, and image parameter settings is also a crucial direction for research. 

Future research should further explore the applications of semi-supervised, weakly supervised, and unsupervised learning methods in NDT techniques. These learning approaches not only provide effective solutions when labeled data are limited but also enhance model robustness and generalization through self-supervised learning, image-level annotation, or unsupervised clustering. Additionally, advancements in feature learning (FL) and representation learning (RL) will be crucial for improving the performance of object detection algorithms [140,141,142]. Future studies should focus on designing more efficient and diverse feature extraction methods, including multimodal data fusion, to better capture semantic information and contextual relationships in images. 

In addition, the application of Generative Adversarial Networks (GANs) in data augmentation suggests that object detection algorithms will increasingly rely on synthetic data to enhance model generalization and robustness against interference. Combining unsupervised learning models with GAN techniques not only addresses the issue of low accuracy due to insufficient sample data but also effectively mitigates the annotation costs associated with large sample datasets, thereby further enhancing the potential applications of related deep learning algorithms in the field of NDT. With a growing emphasis on interpretability, interpretable models will also be a critical focus of future research in object detection algorithms, enhancing model transparency and credibility. Ultimately, interdisciplinary collaboration and technology transfer will facilitate the customized application of object detection algorithms in the inspection and testing of pressure equipment, providing more convenient and efficient solutions for inspection and testing and bringing forth additional opportunities for innovation in engineering and research domains.

## Figures and Tables

**Table 1:** Summary of the application of GAN for data augmentation.

Ref.	Model	Dataset Size after Augmentation	Objective	Performance Metric	Value
[68]	DCGAN	3600 samples	Data augmentation for defect detection and classification using deep learning	Sensitivity	DC: 95.33%
					AC: 92.28%
	ACGAN				Info: 94.06%
				Specificity	DC: 99.16%
	InfoGAN				AC: 98.56%
					Info: 98.81%
[72]	DetectionGAN	200,000 samples	Generate defective ultrasound images for training purposes	Avg subjective grade (1–5)	3.6
	SPADE GAN				3.3
[74]	DetectionGAN	202,278 samples	Generate ultrasonic B-scan images of defects at different locations and improve the performance of the object detection network	AP Training	75.7%
[76]	CECGAN	20,360 samples	Solve the problem of data imbalance and improve the accuracy of defect detection	Defect recognition accuracy	92.5%
[77]	IWFF-GAN	10,703 samples	To improve the accuracy of normal weld recognition	AUC	0.9903
[78]	CVAE-GAN	3500 samples	Reconstruct the missing MFL samples and augment diverse defect sample	RMSE	0.05

**Table 2:** Summary of the application of object detection algorithm combined with NDT in anomaly recognition.

Ref.	Model	NDT Method	Dataset Size	Objective	Performance Metric	Value
[82]	U-Net	MT	3602 samples	Automated detection of the presence or absence of MT defects	Accuracy	85.8%
[4]	U-Net	X-ray	2627 samples	Investigate the effects of signal-to-noise ratio (SNR) and contrast-to-noise ratio (CNR) on the performance of U-net deep learning semantic segmentation model	IoU	High CNR: 0.9594
						Low CNR: 0.5875
[5]	SPP-net	X-ray	16,000 samples	Automatic anti-counterfeiting detection of X-ray welding film	Accuracy	Characters and markers recognition: 91.4%
						Welding seam fingerprint matching: 89.6%
						Overlap regions matching: 87.3%
[89]	Faster R-CNN	X-ray	1665 samples	X-ray defects of different shapes and parts can be detected with high precision	mAP	0.863
[90]	CNNs	DR	1469 samples	Achieve accurate inspection of DR-based casting defect inspection system	Accuracy	91.17%
[91]	GAMSR	DR	3230 samples	Improve the defect detection performance of DR Images of aluminum castings	mAP	0.937
[11]	MBDFN	UT	5608 samples	Identify and classify weld defects	Accuracy	92.2%
[98]	DefectDet	UT	4000 samples	Identifying types and locations of defects from UT images	mAP	0.913
[103]	EFRCNN	TOFD	1913 samples	Locating and identifying five types of defects in TOFD images: porosity, crack, slag inclusion, lack of penetration, lack of fusion	Accuracy	92.35 ± 2.04%
[59]	DCNN	PAUT	Not noted	Classify defects in PAUT welding signals	Accuracy	97%
[106]	YOLOv8_SBA	PAUT	2286 samples	Enhancing the detection accuracy and efficiency of PAUT, identifying and classifying FBH, SDH, and defect-free regions	F1 scores and IoU	FBH F1 scores: 100%
						FBH IoU: 86.04%
						SDH F1 scores: 82.50%
						SDH IoU: 65.96%
[109]	T mR-CNN	PAUT	125 samples	Achieved precise identification of defect characteristics and determination of defect sizes	Accuracy and F1 scores	Accuracy: 91.46%
						F1 scores: 91.62%
[117]	SwinYv5 and CR-CNN	MFL	540 samples	Detect defects in collected MFL signals and estimate defect dimensions based on depth, width, and length	Precision	98.90%
[118]	VDTL	MFL	16,000 samples	Predict MFL defect sizes and estimate defect cross-sectional profiles	Prediction errors	Length error: 0.67 mm
						Depth error: 0.97%
						Profile error: 2.67%
[122]	Mask R-CNN	ECT	320 samples	Detecting weld seam defects in ECT images	Defect size	A groove defect measuring 3 mm (length) × 0.1 mm (width) × 0.5 mm (depth)
						A FBH measuring Φ0.8 mm × 0.5 mm
[123]	ResNeXt1D-38	ECT	48,000 samples	Automated depth assessment of defects on metal surfaces during ECT	Accuracy	93.58%
[63]	ANN	AE	1440 samples	Identifying acoustic emission signals under different conditions and leakage sizes	Accuracy	99.08%
[136]	DNN	AE	90 samples	classify pressure cracks during acoustic emission testing	Average classification accuracy (ACA)	94.67%

## Data Availability

No new data were created or analyzed in this study. Data derived from public domain resources.
